# Long head of the biceps as a local autograft for surgical treatment of high-grade acromioclavicular dislocations: a clinical anatomy and feasibility study

**DOI:** 10.1016/j.xrrt.2025.02.003

**Published:** 2025-03-08

**Authors:** Nuno Sevivas, Mariana Pinto, Ana Catarina Ângelo, Clara Azevedo, Manuel Ribeiro da Silva, Rui Claro, João Espregueira-Mendes, Hélder Pereira

**Affiliations:** a Life and Health Sciences Research Institute (ICVS), School of Medicine, University of Minho, Campus de Gualtar, Braga, Portugal; b ICVS/3B's - PT Government Associate Laboratory, Braga/Guimarães, Portugal; c Shoulder and Elbow Unit, Orthopaedics Department, ULSAM Médio Ave, Famalicão, Portugal; d Shoulder and Elbow Unit, Orthopaedics Department, Trofa Saúde Hospital Braga Sul, Braga, Portugal; e Shoulder and Elbow Unit, Department of Orthopaedic Surgery, Hospital dos SAMS de Lisboa, Lisbon, Portugal; f Shoulder and Elbow Unit, Orthopaedics Department, Joaquim Chaves Saúde, Carcavelos, Portugal; g Shoulder and Elbow Unit, Orthopaedic and Musculoskeletal Centre, Cuf Porto Hospital, Porto, Portugal; h Shoulder and Elbow Unit, Orthopaedics Department, Centro Hospitalar Santo António, Porto, Portugal; i Clínica Espregueira Mendes, Porto, Portugal; j Orthopaedics Department, Centro Hospitalar Póvoa de Varzim/Vila do Conde, Póvoa de Varzim, Portugal; k Ripoll y De Prado Sports Clinic: Murcia-Madrid FIFA Medical Centre of Excellence, Madrid, Spain

**Keywords:** Long head of the biceps, Autograft, Acromioclavicular dislocation, Chronic dislocation, Acromioclavicular ligamentoplasty, Surgical treatment, Clinical anatomy study

High-grade acromioclavicular (AC) dislocations are a major clinical concern owing to their compromised ligament healing capacity, leading to instability, pain, and decreased function. Surgical repair is the treatment of choice, often requiring biological augmentation with a graft, particularly in cases of subacute and chronic injuries. There is no consensus on the definition of acute, subacute, and chronic lesions, and surgical outcomes can be hampered by nonhealing of the ligament and loss of reduction. Furthermore, controversy remains when considering synthetic vs. biological augmentation, and the current role of tissue engineering in several scenarios of orthopedic surgeries.^7^^,^^10^ Biological augmentation using an autograft, particularly by pediculated tendon transfer,^20^ is a promising approach for improving the long-term outcomes of AC dislocations.

Drawing parallels from the treatment of anterior cruciate ligament tears in the knee, where historically the outcomes of synthetic augmentation have raised concerns despite growing interest in tissue engineering approaches,^6^ for many decades, the use of biologic (autologous or allogenic) grafts for ligamentoplasty has been the standard practice, even in acute settings.^11^ Moreover, often, and with growing indications, an extra-articular biological augmentation procedure is added to restore rotational stability and protect against reinjury.^13^^,^^22^

This factor also increased our attention in the search for possibilities of using a local autograft with adequate biomechanical and biological properties, at least in nonacute AC dislocation when surgery is required.

We propose a novel technique for treating AC dislocations using the long head of the biceps (LHB) tendon as an autograft. This method augments the reconstruction of the coracoclavicular (CC) and AC ligaments, restores both vertical and horizontal AC joint stability, and potentially decreases the subsidence and loss of reduction rates associated with AC joint reconstruction techniques using suspensory systems.

## Surgical indications and contraindications

Surgical treatment of AC joint dislocations depends on several factors, namely, the classification of structural injury (evaluated by the degree of separation and, consequently, the severity of ligament injury) and the patient's functional status (activity level).

Low-grade (types I and II in the Rockwood classification) AC dislocations are typically managed conservatively, with favorable clinical outcomes.^8^^,^^23^

Surgical treatment of higher-grade injuries (types IV and V) is usually advised to restore the anatomy, relieve pain, and improve strength and endurance^28^; however, the treatment of type III injuries remains controversial.^5^

The International Society of Arthroscopy, Knee Surgery and Orthopaedic Sports Medicine (ISAKOS) Upper Extremity Committee proposed a modified Rockwood classification, subdividing injuries into grade IIIA (stable) and grade IIIB (unstable) based on the absence or presence of a clavicle overriding the acromion on the cross-body adduction view, indicating an important horizontal instability component. The unstable grade IIIB lesions were proposed to be better treated with surgical treatment.^4^

Finally, contraindications include patients with a low functional status, who benefit less from surgical treatment, and those with infections.

## Treatment options

Both anatomical and nonanatomical AC joint reconstruction techniques provide good postoperative results; however, there is no consensus regarding the optimal reconstruction technique.^32^ In acute injuries, one of the most popular strategies uses a suspensory system with buttons passed through a transclavicular–transcoracoid drilling technique that provides primary dynamic fixation of the reduced joint, relying on the healing potential of the native CC ligaments. There are several other surgical methods of treating acute and chronic AC-joint dislocations including the hook plate fixation^27^^,^^12^^,^^31^ and circumferential sutures cerclage techniques.^3^^,^^18^^,^^24^^,^^29^

Hook plate fixation is an effective treatment option that achieves good functional outcome scores but necessitates a second surgery for implant removal. Moreover, subacromial erosion was observed in 40% of patients, although this seems not to affect long-term pain.^15^ Nevertheless, other techniques, such as suture button fixation, are currently preferred, as they have been associated with better functional outcomes and lower pain scores.^30^

However, in high-grade injuries, it is advisable to restore horizontal stability through a procedure that reinforces the AC capsule and ligaments.^1^^,^^2^^,^^3^^,^^18^ CC reconstruction with additional AC cerclage demonstrated good clinical results, despite failing to prevent an increase in CC distance over time as observed radiographically.^24^ However, AC distances are generally lower when an additional AC cerclage is used.^29^

Finally, biological augmentation is mandatory in nonacute settings and can be useful in subacute settings.

## Novel technique

1. Positioning: The patient was placed in a beach chair position with the affected limb at the side, optionally held using a mechanical arm holder (Fig. 1).

**Figure 1:**
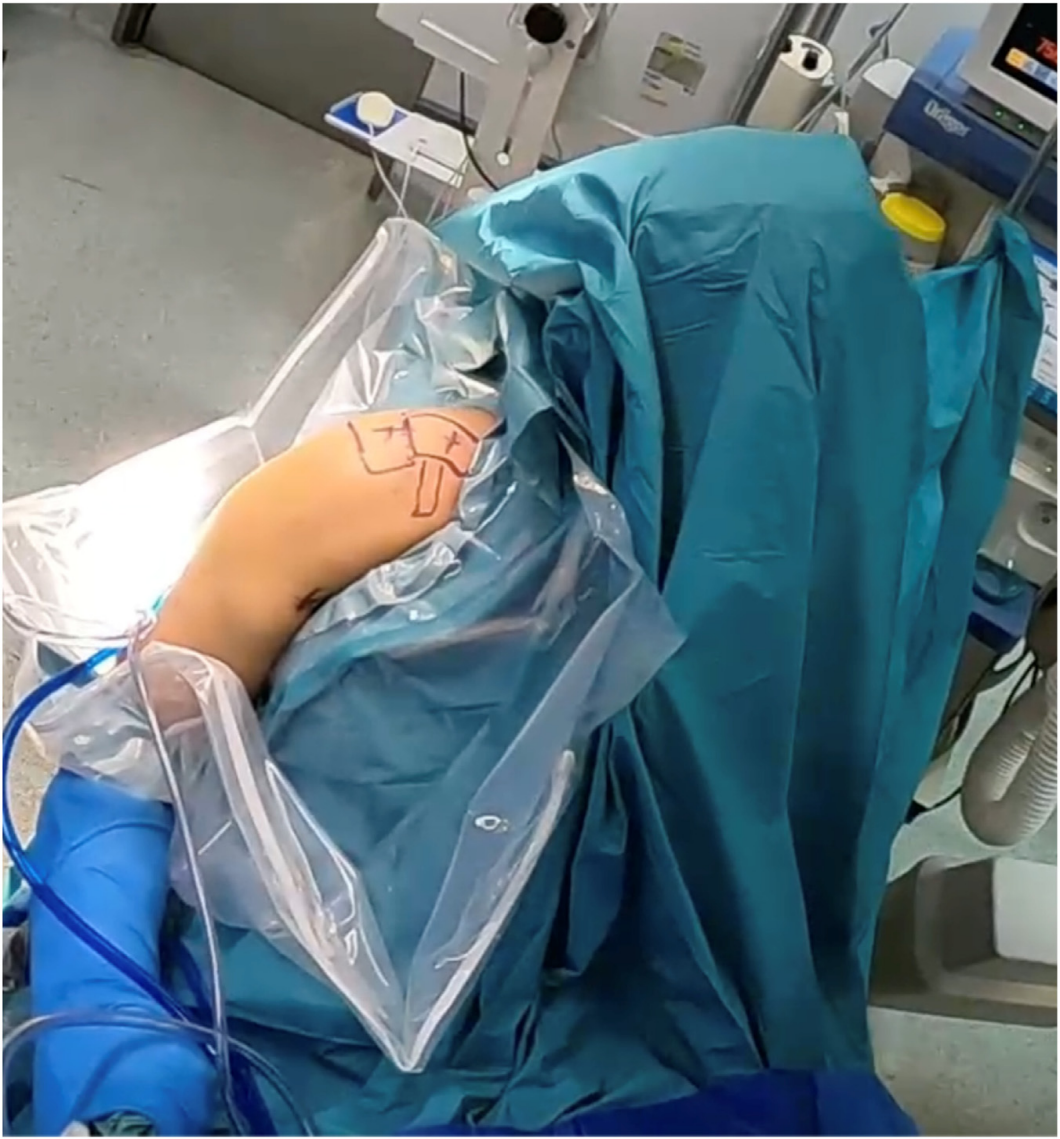
The patient is placed in the beach chair position with the affected limb at the side, optionally held by a mechanical arm holder.
2. Approach: The deltopectoral approach was extended proximally, passing above the coracoid process and clavicle, approximately 3 cm medial to the AC joint. The approach was also extended distally to expose the subpectoral region, providing clear visualization of the LHB myotendinous junction
3. LHB tenotomy and subpectoral tenodesis: The LHB was tenotomized 1 cm proximal to the myotendinous junction and underwent tenodesis with an all-suture anchor (Y-Knot RC 2.8 mm; Conmed, Largo, FL, USA), as per the technique described by Scully et al.^25^ The remnant distal stump of the tendon was prepared using two locked Krackow stitches. The free ends of the sutures were used to shuttle the tendon down to the anchor and were tied over to secure the tendon to the anterior humeral cortex (Fig. 2).

**Figure 2:**
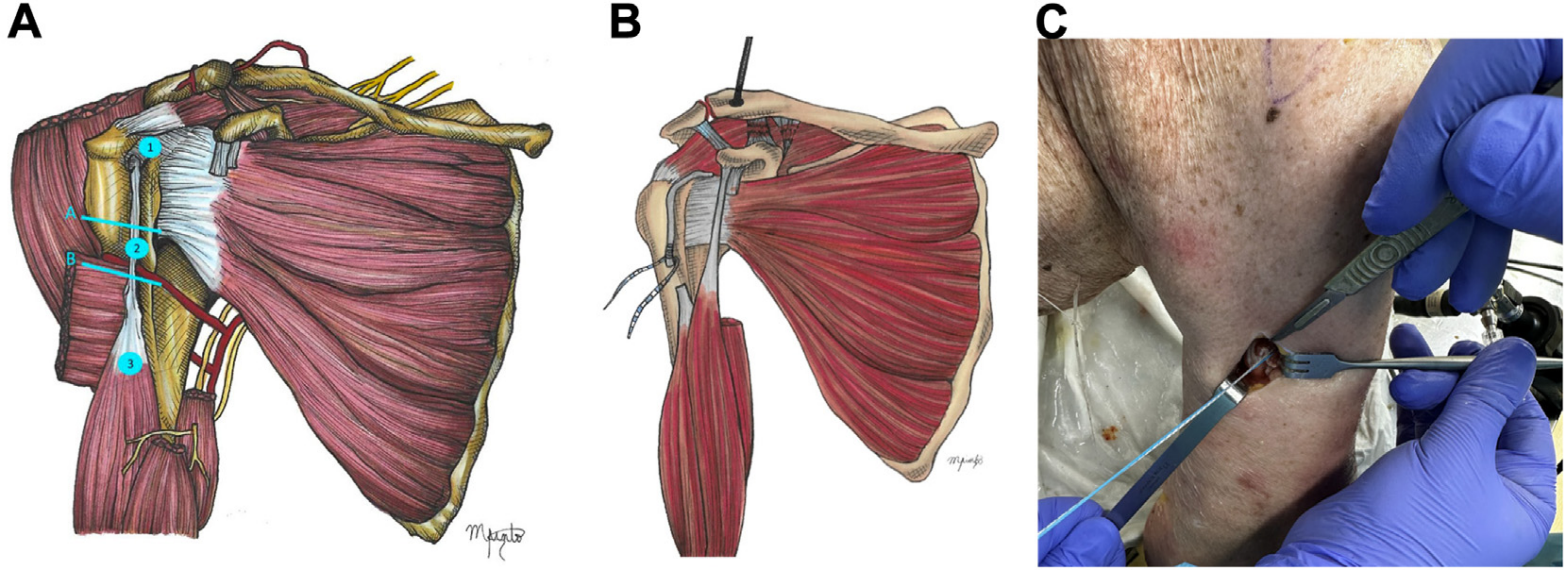
LHB tenotomy and subpectoral tenodesis: (**A**) The LHB is tenotomized 1 cm proximal to the myotendinous junction (near zone 3 in this image). (**B**) The proximal stump of the LHB graft was rerouted proximally, maintaining its original insertion into the supraglenoid tubercle and superior labrum. (**C**) The distal stump of LHB is tenodesed with an all-suture anchor (Y-Knot RC 2.8 mm; Conmed, Largo, FL, USA). *LHB*, long head of the biceps.
4. Rotator interval opening: The fascia delimiting the anterior border of the bicipital groove and the rotator interval were opened. The space below the coracoid process was cleaned for proper visualization.
5. Graft preparation and passage: The proximal stump of the LHB graft was rerouted proximally, maintaining its original insertion in the supraglenoid tubercle and superior labrum, and was retrieved through the rotator interval. Based on our anatomical studies, the expected length of the LHB tendon graft is 10 cm, measured from its origin at the supraglenoid tubercle to the tenotomized distal stump. The tendon diameter was verified using a calibrated sizer and trimmed to 4-4.5 cm. The prepared tendon was then secured with two sutures in a Krackow fashion (Fig. 3).

**Figure 3:**
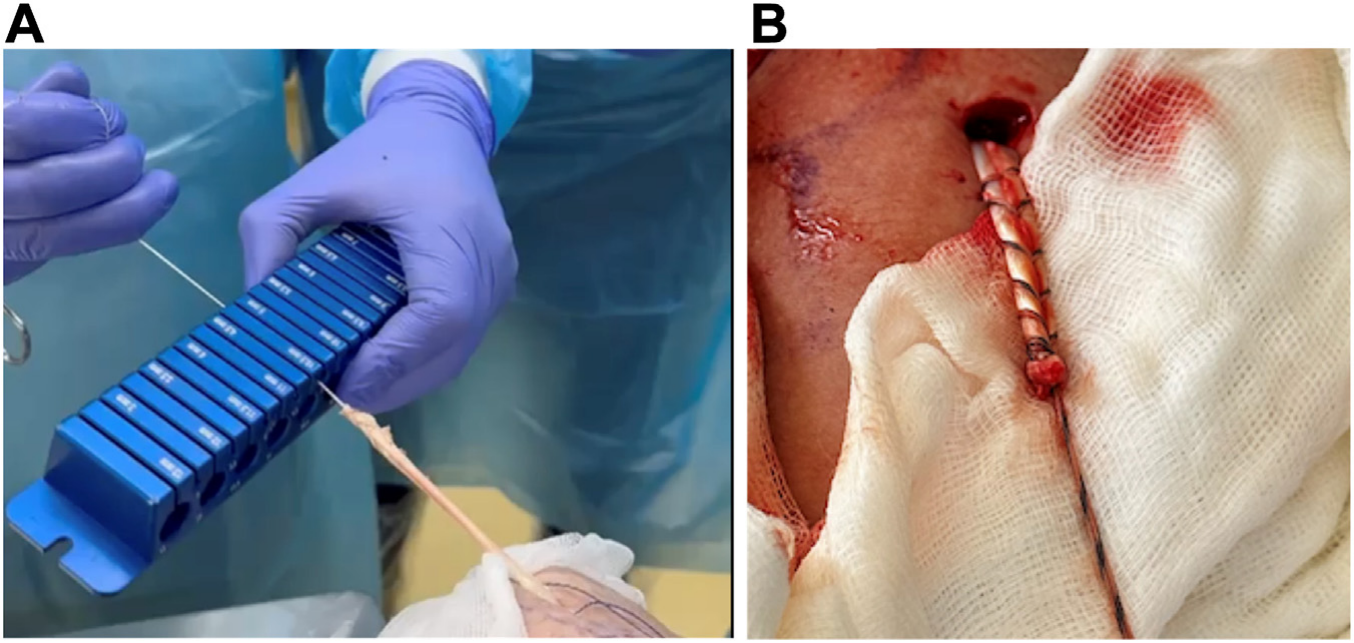
LHB graft preparation: (**A**) The proximal stump of the LHB graft is rerouted proximally, maintaining its original insertion in the supraglenoid tubercle and superior labrum, and retrieved through the RI. The tendon diameter is verified using a calibrated sizer and trimmed to 4-4.5 cm. (**B**) The prepared tendon was then secured with two sutures in a *Krackow* fashion. *LHB*, long head of the bicep; *RI*, rotator interval.
6. CC tunnel: A 2.4 mm guide wire was inserted, starting at the superior border of the clavicle (3.5 cm medial to the AC joint) and sequentially exiting at the inferior border of the clavicle, the superior border of the coracoid process, and finally the midpoint of its inferior border. This was performed using a drill guide (Conmed Infinity anterior cruciate ligament/posterior cruciate ligament Femoral Footprint Guide Arm, Infinity Guide Body, Infinity Guide Sleeve Straight 2.4 mm; Conmed, Largo, FL, USA) at an angulation of 70°-80°. Fluoroscopic confirmation was then obtained before creating the tunnel with a 4.5 mm drill (Fig. 4).

**Figure 4:**
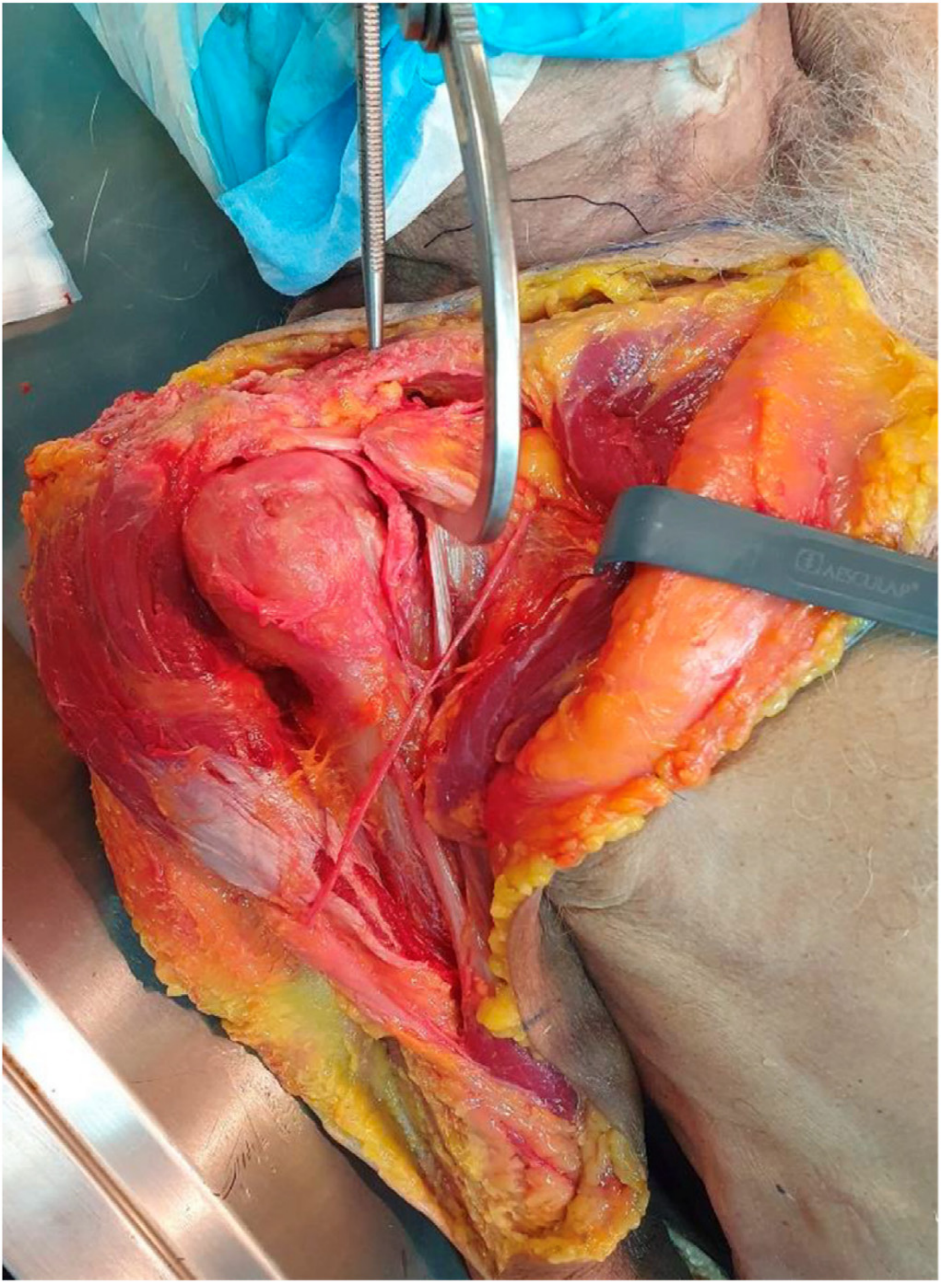
Coracoclavicular (CC) tunnel: A 4.5 mm tunnel is drilled, connecting the superior border of the clavicle (3.5 cm medial to the AC joint) and exiting at the inferior border of the coracoid process using a drill guide. A 2.4 mm guide wire is then passed, ideally at the midpoint of the coracoid process, and fluoroscopic confirmation is obtained before creating the tunnel with a 4.5 mm drill. *AC*, acromioclavicular.
7. CC graft passage and fixation: A suture passer (Super Shuttle; Conmed, Largo, FL, USA) was used to shuttle both the suture strands, holding the free end of the tendon, and the suspensory system (Infinity Button; Conmed, Largo, FL, USA), through the tunnel. The AC joint was reduced, and the system was locked with the superior button apposed to the upper border of the clavicle, providing vertical stability (Fig. 5).

**Figure 5:**
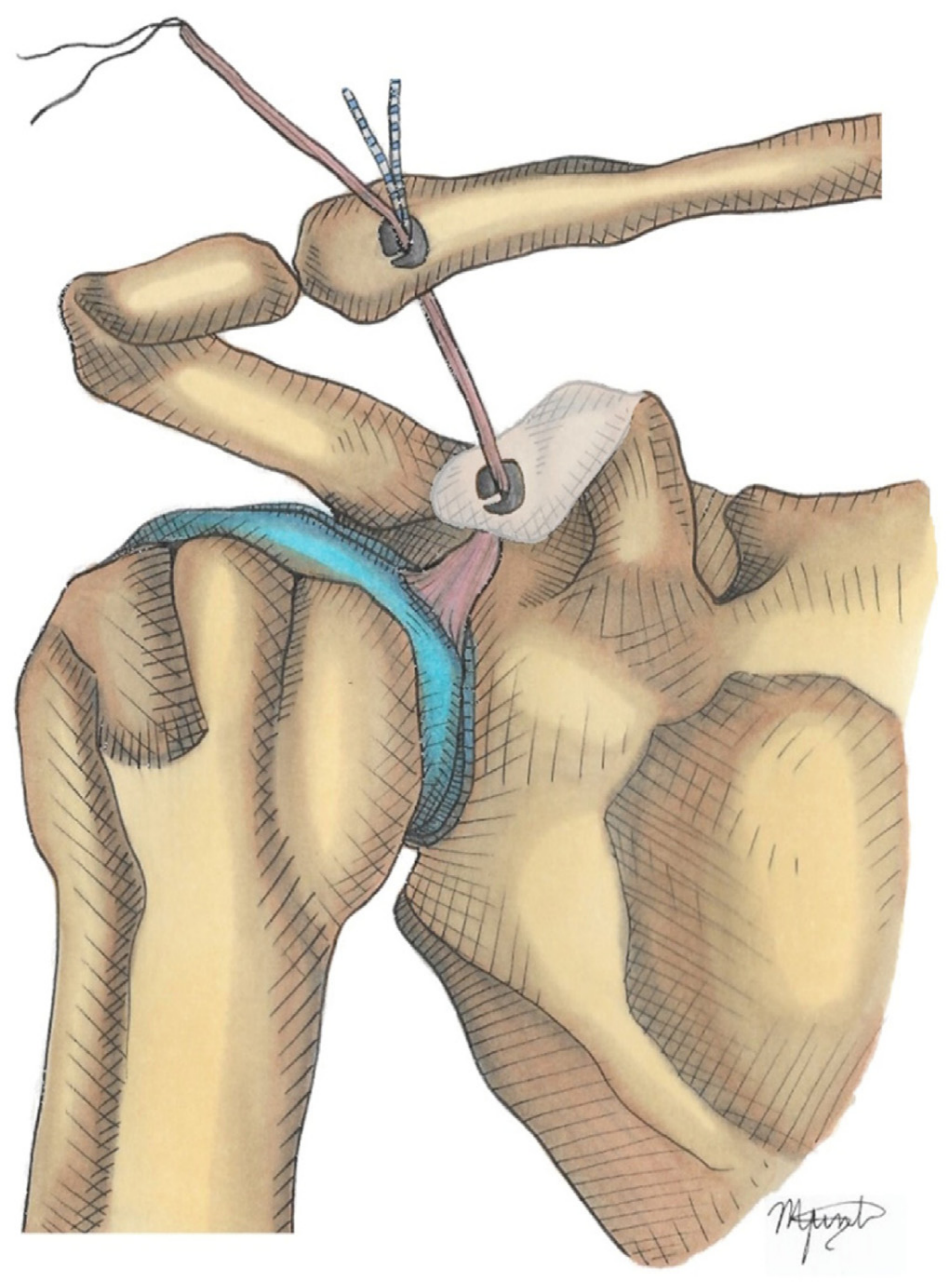
CC graft passage and fixation: A suture passer is used to shuttle both suture strands, holding the free end of the tendon and suspensory system through the tunnel. The AC joint was reduced, and the system was locked with the superior button apposed to the upper border of the clavicle, providing vertical stability. *CC*, coracoclavicular; *AC*, acromioclavicular.
8. Acromion tunnel and fixation: One-centimeter lateral to the AC joint, a 3 mm tunnel parallel to the joint line was drilled in the acromion. The remaining LHB tendon was passed posteriorly to anteriorly and fixed using a knotless anchor (2.8 or 3.3 mm Poplok; Conmed, Largo, FL, USA). If possible, the posterosuperior ligament and capsule of the AC joint were reinforced with side-to-side stitches to the graft (Fig. 6).

**Figure 6:**
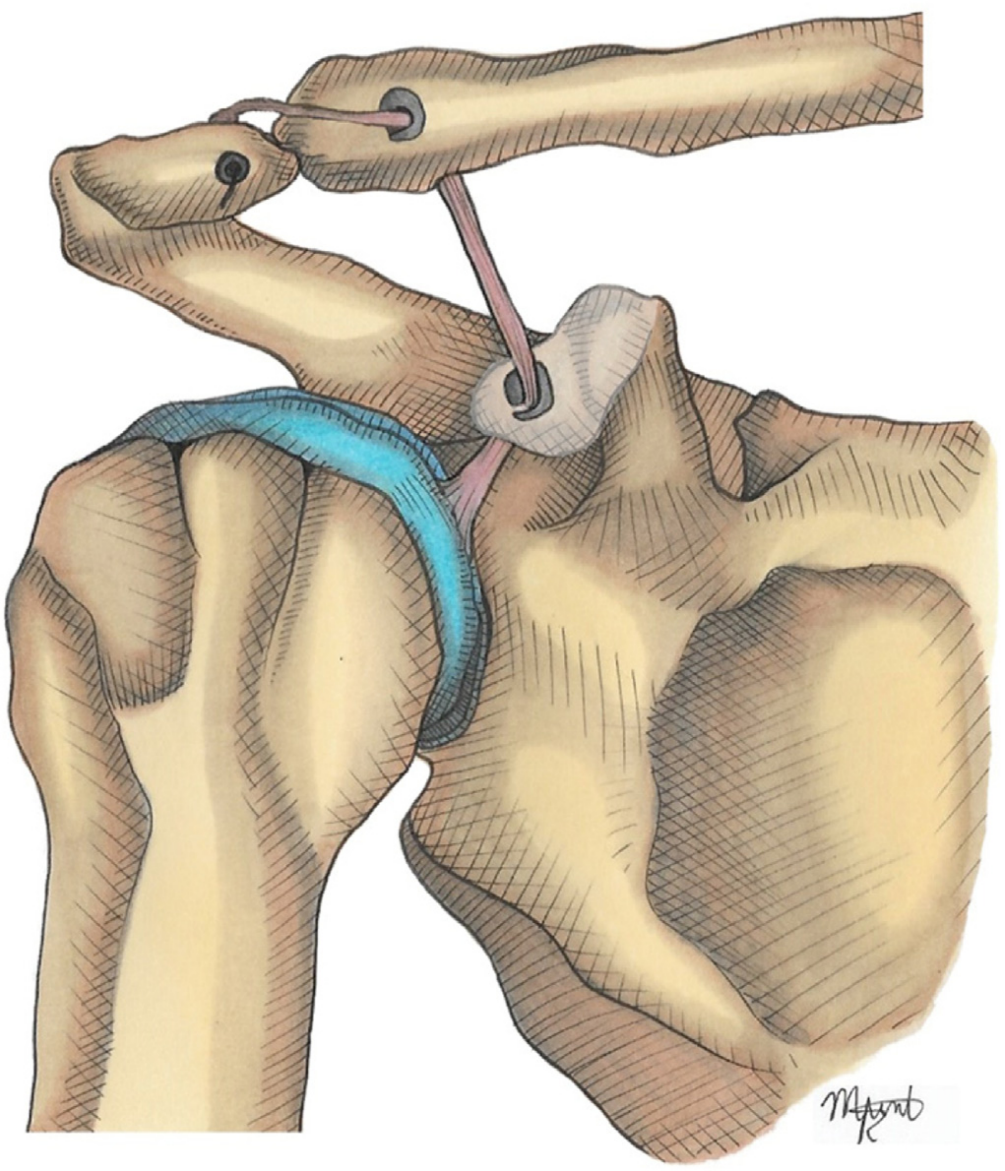
Acromion tunnel and fixation: 1 cm laterally to the AC joint, a 3 mm tunnel parallel to the joint line is drilled in the acromion. The remaining LHB tendon is passed from posterior to anterior and fixed using a Knotless anchor. If possible, the AC joint posterosuperior ligament and capsule are reinforced with side-to-side stitches to the graft. *LHB*, long head of the bicep; *AC*, acromioclavicular.

## Postoperative rehabilitation

After the procedure, and following a Lacheta et al^17^ based protocol, we recommend sling immobilization for 4 weeks, allowing immediate active-assisted and passive range of motion exercises limited to 90° of shoulder flexion. Active exercises should begin 4 weeks postsurgery and full range of motion is allowed. Resisted elbow flexion and forearm supination are restricted for 6 weeks. Overhead strengthening exercises and heavy lifting should be avoided for at least 3 months.

## Advantages of LHB autograft ligamentoplasty

• Biomechanical and biological superiority: The biological augmentation provided by the LHB autograft, which maintains the original insertion at the supraglenoid tubercle, potentially improves outcomes for both chronic and acute AC dislocations.
• Anatomical location: Located near the injured AC joint, it prevents donor site morbidity in an uninjured limb and at a distance, while maintaining its original insertion in the supraglenoid tubercle. The graft only needs to heal in the clavicle because the scapular connection remains the original.
• Graft diameter and length: The biomechanical properties of ligamentoplasty are enhanced by the greater diameter and length of the graft in comparison with the CC ligament, which is used classically with Weaver–Dunn ligamentoplasty.
• Stabilization: Increased vertical and horizontal AC joint stability, potentially reducing the loss rate of reduction and subsidence in the long term.

## Outcomes of the technique

Using grafts to biologically augment ligament reconstruction in cases of AC dislocation can improve postoperative clinical and radiological outcomes. Potential donor site morbidity associated with the use of popular autografts, such as hamstrings, must be considered and explained to the patients. LHB has been used as an autograft for several shoulder treatment modalities.^9^^,^^19^^,^^20^^,^^26^

Utilizing the LHB tendon as a graft for AC and CC ligament, reconstruction has several advantages. First, its anatomical proximity to the AC joint complex allows for preserving its vascular supply, as the tendon's insertion into the scapula (supraglenoid tubercle) remains intact. This enhances the healing and ligamentization processes by requiring only integration at the clavicle, supporting better overall outcomes. Moreover, as a local autograft, it avoids donor-site morbidity in the uninjured limb.

This novel technique demonstrates the anatomical feasibility of using LHB as an autograft for the surgical treatment of high-grade AC dislocation. This technique is performed using an open approach and allows biological augmentation of both CC and AC reconstructions, thus addressing vertical and horizontal instability.

## Disadvantages

Potential complications related to this procedure may arise during subpectoral LHB tenodesis at low rates and includes neuropathy, persistent bicipital pain or deformity (Popeye sign).^21^ Another theoretical risk involves tunnels drilled through the coracoid process, clavicle, and acromion, and passage of the graft through these tunnels. The most significant risk is the potential for intraoperative breach of the medial or lateral cortex of the coracoid process, which can lead to fracture. This risk is heightened by the challenge of achieving accurate anatomical tunnel placement using the transclavicular–transcoracoid drilling technique,^16^ compounded by the requirement for a 4.5 mm drill hole.

Moreover, other theoretical disadvantages may be related to an open approach and aggressiveness. Arthroscopic or arthroscopy-assisted techniques can minimize these complications. In future studies, we aim to prove the feasibility of arthroscopically performing this technique.

Other possible limitations include harvest-site morbidity, increased surgical time, and the absence of LHB. Finally, the technique can be viewed as adding complexity; however, when comparing similar alternatives with ligamentoplasty, it seems to be easier to perform and avoids the morbidity of harvesting a graft in the lower limb, which can have a rate as high as 60%, including sensory deficits and deep venous thrombosis.^14^

Moreover, theoretical concerns can be raised about the tension on the superior labrum with this technique and its potential as a source of pain. It is important to note that painful symptoms associated with superior labrum from anterior to posterior tears are often attributed to dynamic traction of the LHB tendon during eccentric contraction of the biceps muscle. This unfavorable mechanical condition is effectively eliminated with subpectoral biceps tenodesis in our technique. Notably, none of our clinical cases have reported intra-articular shoulder pain to date.

Finally, it is advisable to underline that a continuous follow-up will be essential in assessing the durability of the graft function and the potential for complications at long-term.

## Conclusion and future perspectives

The main finding of this study was that coracoclavicular and AC reconstruction using LHB as a local autograft is feasible using the proposed technique.

AC joint injuries are common and are often underestimated. High-grade chronic AC joint injuries can compromise the natural ligament healing and cause persistent pain, instability, and reduced function. Surgical interventions involving various anatomical and nonanatomical reconstruction techniques have provided significant postoperative improvements; however, the optimal technique is still debatable.^5^

Using the LHB tendon as an autograft to augment AC joint reconstruction is a promising technique offering a logical and innovative solution that warrants further investigation. This novel method provides enhanced biomechanical and biological properties, potentially improving the treatment outcomes of high-grade AC dislocation. Future studies, both in cadaveric and clinical settings, should aim to demonstrate the feasibility of an endoscopic approach, objective biomechanical improvement, and evaluation of clinical outcomes against other graft options.

## Acknowledgment

The authors would like to thank Editage (www.editage.com) for English language editing.

## Disclaimers:

Funding: No funding was disclosed by the authors.

Conflicts of interest: The authors, their immediate families, and any research foundation with which they are affiliated have not received any financial payments or other benefits from any commercial entity related to the subject of this article.
